# Development and Characterization of Fully Renewable and Biodegradable Polyhydroxyalkanoate Blends with Improved Thermoformability

**DOI:** 10.3390/polym14132527

**Published:** 2022-06-21

**Authors:** Patricia Feijoo, Kerly Samaniego-Aguilar, Estefanía Sánchez-Safont, Sergio Torres-Giner, Jose M. Lagaron, Jose Gamez-Perez, Luis Cabedo

**Affiliations:** 1 Polymers and Advanced Materials Group (PIMA), Universitat Jaume I (UJI), Avenida de Vicent Sos Baynat s/n, 12071 Castelló, Spain; pfeijoo@uji.es (P.F.); samanieg@uji.es (K.S.-A.); esafont@uji.es (E.S.-S.); gamez@uji.es (J.G.-P.); 2 Novel Materials and Nanotechnology Group, Institute of Agrochemistry and Food Technology (IATA), Spanish National Research Council (CSIC), Calle Catedrático Agustín Escardino Benlloch 7, 46980 Paterna, Spain; storresginer@upv.es (S.T.-G.); lagaron@iata.csic.es (J.M.L.)

**Keywords:** poly(3-hydroxybutyrate-*co*-3-valerate), poly(3-hydroxybutyrate-*co*-3-hydroxyhexanoate), biopolymer blends, crystallinity, thermoforming, food trays

## Abstract

Poly(3-hydroxybutyrate-*co*-3-valerate) (PHBV), being one of the most studied and commercially available polyhydroxyalkanoates (PHAs), presents an intrinsic brittleness and narrow processing window that currently hinders its use in several plastic applications. The aim of this study was to develop a biodegradable PHA-based blend by combining PHBV with poly(3-hydroxybutyrate-*co*-3-hydroxyhexanoate) (PHBH), another copolyester of the PHA family that shows a more ductile behavior. Blends of PHBV with 20% wt., 30% wt., and 40% wt. of PHBH were obtained by melt mixing, processed by cast extrusion in the form of films, and characterized in terms of their morphology, crystallization behavior, thermal stability, mechanical properties, and thermoformability. Full miscibility of both biopolymers was observed in the amorphous phase due to the presence of a single delta peak, ranging from 4.5 °C to 13.7 °C. Moreover, the incorporation of PHBH hindered the crystallization process of PHBV by decreasing the spherulite growth rate from 1.0 µm/min to 0.3 µm/min. However, for the entire composition range studied, the high brittleness of the resulting materials remained since the presence of PHBH did not prevent the PHBV crystalline phase from governing the mechanical behavior of the blend. Interestingly, the addition of PHBH greatly improved the thermoformability by widening the processing window of PHBV by 7 s, as a result of the increase in the melt strength of the blends even for the lowest PHBH content.

## 1. Introduction

Plastics are key materials in our daily lives, growing every year both in presence and relevance. At the same time, there is also a growing concern about the negative environmental impact they cause throughout their whole lifecycle: from their origin and production to their eventual disposal [1], making their correct waste management of the utmost importance. Moreover, around 46% of global plastic waste comes from the food-packaging sector and single-use products, with lifetimes that are generally just a few weeks and even days in some cases [2].

Therefore, food-packaging products result in a considerable and continuous source of domestic plastic waste. Plastic materials generally exhibit high chemical stability and inertness, both characteristics of interest in the packaging sector, but this, in turn, results in a high permanence and accumulation of their residues, thus rendering an environmental problem when they are not correctly managed [3,4,5]. In this regard, the plastic-pollution concern has led to authorities implementing restrictive policies and regulations for banning single-use products and bags [5,6]. As a consequence, the development of alternative materials to replace commodities currently represents a priority for both industry and academia. In this regard, bioplastics became an interesting option for this target due to their sustainable and eco-friendly attributes [6].

In this context, polyhydroxyalkanoates (PHAs) are one of the most promising bioplastics because of their triple *bio*-advantages: *bio*-based, *bio*degradable, and *bio*compatibility. PHAs are a family of thermoplastic biopolyesters, naturally synthesized as an intracellular storage product by microorganisms under certain adverse feeding conditions [7]. PHA-producing microorganisms can be fed with pure and controlled feedstock, as well as organic waste or by-products from the agri-food sector (i.e., cheese whey, coffee grounds, etc.), thus rendering a fully renewable, low carbon-footprint material, while contributing to the promotion of the so-called Circular Bioeconomy [8,9]. On the other hand, PHAs have demonstrated to be fully degradable by microorganisms in all aerobic- and anaerobic-relevant environments (industrial compost, home compost, soil, seawater, landfill) at a fast rate, thus being defined as biodegradable materials in accordance with international standards [10]. Furthermore, biocompatibility makes them suitable for medical applications such as tissue engineering or drug delivery [11,12].

Since their first observation by Lemoigne in 1926 [13], more than 150 monomers featuring in PHA alone (homopolymers) or in combination (copolymers) have been reported [14]. However, only a few of these biopolyesters are commercially available, and, among them, the most extended ones are poly(3-hydroxybutyrate) (PHB), poly(3-hydroxybutyrate-*co*-4-hydroxybutyrate) (P3HB-*co*-4HB), poly(3-hydroxybutyrate-*co*-3-hydroxyhexanoate) (PHBH), and poly(3-hydroxybutyrate-*co*-3-hydroxyvalerate) (PHBV) [15]. The physical and chemical properties of PHAs range broadly depending on the structure of their constituent monomers. Based on the chain length (CL) of the monomers they are made from, PHAs can be categorized into three groups: *short* (SCL-PHA, 3-5 carbon atoms), *medium* (MCL-PHA, 6-14 C), and *long* (LCL-PHA, >15 C). Generally, SCL-PHAs are rigid, brittle, and high-crystalline thermoplastics, while MCL- and LCL-PHAs are flexible, ductile, and show lower crystallinity [16].

PHBV is an SCL-PHA with high crystallinity. It possesses a good mechanical performance in terms of stiffness and strength, being comparable to polypropylene (PP), and having good barrier properties similar to polyethylene terephthalate (PET) [17]. In order to improve the thermal stability and allow better processability, copolymers with different contents of 3-hydroxyvalerate (3HV) are produced. However, both PHB and PHBV polymers with low 3HV contents present a major weakness for plastic applications: an intrinsic fragility that increases over time due to a second crystallization and physical ageing phenomenon [18]. Furthermore, their high crystallinity results in a narrow processing window, thus hindering their processability in conventional industrial equipment [19].

A great deal of effort has been devoted to overcoming the shortcomings of PHB and PHBV for plastic applications, highlighting blending with other resins as a useful way of obtaining new materials with improved properties and compatible with industrial processes [20]. Works with both biodegradable [polylactide (PLA) [21], poly(butylene adipate terephthalate) (PBAT) [22], poly(*ε*-caprolactone) (PCL) [23], poly(propylene carbonate) (PPC) [24], and poly(butylene succinate) (PBS) [25]] as well as non-biodegradable polymers [such as poly(ethylene-*co*-vinyl acetate) (EVA) [26], polyethylene oxide (PEO) [27], and thermoplastic polyurethane (TPU) [28]] can be found in the literature. The polymer blends render materials with enhanced properties in terms of flexibility, processing temperature window, or toughness. However, all of these blends are immiscible and, therefore, the mixing conditions are a key factor to ensure a good interaction between the two polymers in the blend. For instance, Phua et al. [29] blended PHBV with PBS and achieved no improvement in the mechanical properties. In these cases, the use of compatibilizing agents may help to generate a convenient morphology, resulting in an improvement in the mechanical performance.

In this sense, previous works of the group regarding PHBV blended with TPU found that it was necessary to use epoxy-based reactive agents (TGIC or Joncryl^®^) to improve the elongation at break and toughness without compromising tensile strength and modulus of elasticity [28,30]. The original drop-in-matrix morphology was modified to lower the droplet size and lower ligament thickness, with the addition of compatibilizing agents in blends with TPU contents >20%.

However, blending PHA with other polymers (even if biodegradable), and the use of compatibilizers, could limit the biodegradability of the so-obtained blend in some environments in which the biopolymer was originally biodegradable [31,32]. For instance, the biodegradability of PHBV/PPC blends in soil was restricted to the PHBV fraction, in accordance with the results reported by Tao et al. [33]. Weng et al. [34] also found that the biodegradability of PHBV/PLA mulch films was limited to thermophilic-composting conditions due to the presence of PLA. In seawater, a PHB/PBAT bag showed no relevant marine biodegradation, whereas neat PHB fully degraded [35]. Therefore, the biodegradability of PHBV in a wide range of environments should be preserved.

For this, PHBH will be an interesting candidate since this MCL-PHA is a random copolymer of 3HB and 3-hydroxyhexanoate (3HH) units that possesses a ductile nature and a wider processing window compared to the homopolymer PHB [36]. Due to its low crystallinity, PHBH shows excellent flexibility and elongation at break, but a low Young’s modulus and tensile strength [36,37]. This strategy has been previously used by Nerkar et al. [38] by blending PHB with a different MCL-PHA, poly(3-hydroxyoctanoate) (PHO). Lower crystallinity and enhanced mechanical properties were obtained, which were even better after adding a cross-linking peroxide initiator during processing. More recently, Frone et al. [39] also succeeded by using 10% wt. to 20% wt. of own-biosynthesized PHO, as a toughening agent for PHB, while preserving the biodegradability and biocompatibility of the material.

From the above, this study aims to ascertain the properties of PHBV/PHBH blends as well as SCL- and MCL-PHAs with *a priori* good miscibility but opposite properties. These novel PHA blends represent a convenient approach to improving the mechanical properties and the processability of the PHA biopolymers, without compromising their biodegradability. To this end, three different PHBV:PHBH compositions in % wt. (80:20, 70:30, 60:40) were produced, characterized, and compared to the neat biopolymers. The effect of increasing the PHBH content on the morphology, crystallization, processability, thermal, and mechanical properties of PHBV was analyzed. The characterization of the materials was carried out by means of scanning electron microscopy (SEM), differential scanning calorimetry (DSC), wide-angle X-ray scattering (WAXS), polarized optical microscopy (POM), dynamic mechanical analysis (DMA), melt-flow index (MFI), and thermogravimetrical analysis (TGA). The mechanical performance was evaluated by tensile, tearing, and thermoformability tests. Additionally, the preservation of biodegradability in a marine environment was also corroborated.

## 2. Materials and Methods

### 2.1. Materials

ENMAT 1000P PHBV was purchased from Tianan Biologic Material Co. (Ningbo, China) in pellet form (3-% mol. 3HV content, melting point 170–176 °C, density 1.23 g/cm^3^). X151A PHBH was supplied by the Kaneka Corporation (Osaka, Japan) in pellet form (11-% mol. 3HH content, melting point 126 °C, density 1.19 g/cm^3^). The solvent 2,2,2-trifluoroethanol (TFE) (synthesis grade, density 1.389 g/cm^3^) was acquired from Sigma-Aldrich S.A. (Madrid, Spain) and used for polarized optical microscopy (POM)-sample preparation. Purified alpha-cellulose fiber, grade TC40 (cellulose content > 99.6%) from CreaFill Fibers Corp. (Chestertown, MD, USA), was used as the reference material for the marine-biodegradation test. Natural seawater for biodegradation study was kindly provided by the Institute of Environment and Marine Science Research (IMEDMAR-UCV) from the Catholic University of Valencia located in Calpe (Alicante, Spain). The seawater was enriched with ammonium chloride (NH_4_Cl, purity ≥ 99.5%) and potassium dihydrogen phosphate (KH_2_PO_4_, purity ≥ 99.0%), in accordance with ASTM D 6691 recommendations. Both reagents were purchased from Sigma-Aldrich S.A. and used as received.

### 2.2. Sample Preparation

**Figure 1:**
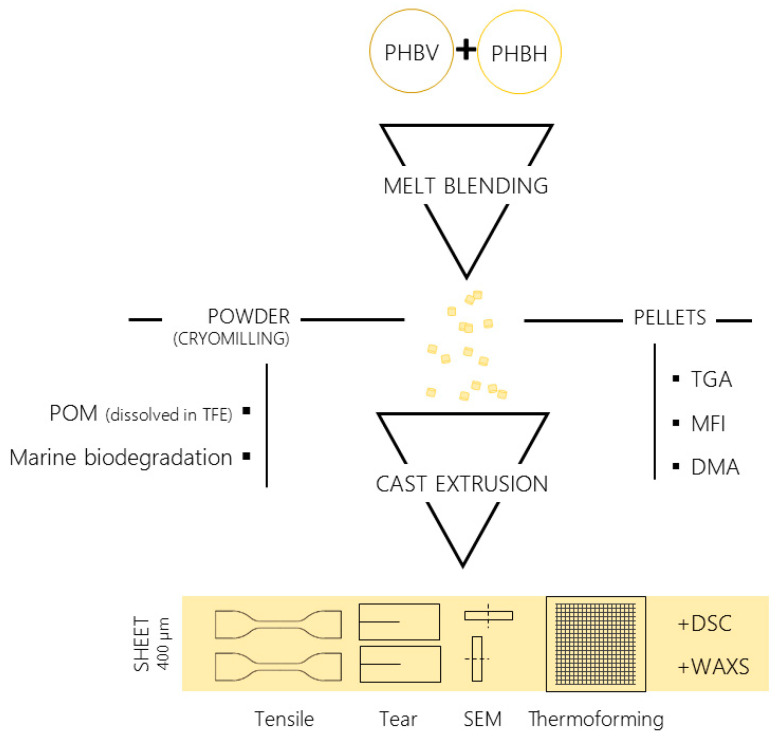
Scheme of sample preparation and characterization carried out.

A scheme of sample preparation can be found in Figure 1. From 100% PHBV to 100% PHBH, three blends of a decreasing PHBV/PHBH weight ratio were prepared in a Haisin TSE-20B (Nanjing, Jiangsu, China) co-rotating twin-screw extruder (Ø = 22 mm, L/D ratio = 32–44). Prior to the blending step, the biopolymers used in this study were dried at 60 °C for at least 8 h in a Piovan DPA 10 dehumidifying dryer (Santa Maria di Sala, VE, Italy). The temperature profile of the extruder was set at 185 °C/175 °C/170 °C/170 °C/170 °C/170 °C/175 °C/180 °C from hopper to nozzle, resulting in a melt temperature at the die of 172–173 °C, and the rotation speed was 200 rpm. All the components were manually dry-mixed before extrusion. The extruded material was cooled in a water bath and pelletized.

For subsequent cast extrusion, all of the PHA pellets were dried at 60 °C for at least 4 h in a dehumidifier (Piovan DPA 10). Each sample was cast extruded into a 400 µm sheet using a single-screw extruder Rheomix 3000P ThermoHaake (ThermoFisher, Karlsruhe, Germany) with a calendering system. The temperature profile was set at 140 °C/165 °C/175 °C/170 °C from hopper to fishtail nozzle, the rotation speed was 100 rpm, and the temperature of the calendering chilling rolls was 40 °C. According to the melt-temperature sensor during extrusion, the polymers were always at a temperature range of 162–165 °C. In spite of its much lower melting point, neat PHBH was processed under identical conditions as blends and PHBV for the sake of comparison. Table 1 summarizes the compositions studied. Samples were named as V for PHBV and H for PHBH, followed by the content of each component as a percentage.

### 2.3. Characterization and Analysis

#### 2.3.1. Scanning Electron Microscopy (SEM)

The 400-µm sheet samples were fractured in both directions (MD and TD) under liquid nitrogen to avoid plastic deformation of the polymer matrix. Prior to SEM analysis, the fractured surfaces were coated by sputtering with a thin layer of platinum for 15 s (equivalent to 2–3 nm) under argon atmosphere using a BalTec500 coater (Pfäffikon, Switzerland). SEM micrographs were obtained by using a high-resolution field-emission JEOL 7001F microscope (Peabody, MA, USA) at a voltage of 5 kV.

#### 2.3.2. Differential Scanning Calorimetry (DSC)

DSC experiments were carried out on a Mettler-Toledo DSC 2 model (Columbus, OH, USA), calibrated with Indium and sapphire standards before use. It was performed in an inert nitrogen atmosphere with a flow-rate of 50 mL/min, with samples of a typical weight of 6 mg from the extruded films in standard 40-µL aluminum crucibles. PHBV and its blends with PHBH were first heated from 25 °C to 190 °C at 40 °C/min and maintained for 2 min to erase thermal history, cooled down to −20 °C at 10 °C/min, and subsequently heated to 190 °C at 10 °C/min. PHBH followed the same cycle with the heating scan up to 165 °C. The melting temperature (T_m_) and enthalpy (∆H_m_), as well as the crystallization temperature (T_c_) and enthalpy (∆H_c_), were calculated from the second heating and cooling curves, respectively.

The melting enthalpies were normalized (∆H_m.N_) to a PHBV weight fraction in the blend and compared with the theoretical melting enthalpy of 100% crystallized PHB, reported to be 146 J/g [40]. This value is commonly considered a good approximation for this particular PHBV, due to its low 3HV content [41].

#### 2.3.3. Wide-Angle X-ray Scattering (WAXS)

WAXS experiments were carried out on the studied samples using a Bruker AXS D4 Endeavor diffractometer (Billerica, MA, USA). Radial scans of intensity versus scattering angle (2*θ*) were recorded at room temperature in the range of 2–40° (2*θ*) (step size = 0.02° (2*θ*), scanning rate = 4 s/step) with filtered CuK*α* radiation (*λ* = 1.54 Å), an operating voltage of 40 kV, and a filament current of 40 mA. The degree of crystallinity (*X_C_*) was calculated after the deconvolution of peaks from diffractograms using Equation (1) [42]:

(1) $$X_{C}\left(\%\right)=\left(\frac{A_{C}}{A_{C}+A_{a}}\right)\cdot 100$$

where *A_c_* represents the total crystalline area of peaks, and *A_a_* is the area of amorphous halo.

#### 2.3.4. Polarized Optical Microscopy (POM)

Sample solutions of 5% wt./vol were prepared by stirring magnetically powdered PHBV, PHBH, and their blends in TFE at room temperature for 24 h, and sonicating for 1 h until the powder was completely dissolved. These solutions, previously filtered by a 0.22 µm PVDF filter, were cast onto a glass coverslip at 50 °C for 5 min and finally dried for 24 h at 40 °C in a vacuum to eliminate residual solvent. The crystallization process was observed using an Olympus BX51 polarized microscopy (Tokyo, Japan) with a 10x times objective and equipped with a Linkam THMS600 hot stage (Salfords, United Kingdom). Cast samples were heated up to 190 °C and maintained for 2 min to ensure complete melting. Then, they were quenched at a cooling rate of 100 °C/min to 100 °C and maintained for 15 min. This temperature cycle was repeated for a second time. Images were taken with a digital camera attached to the objective and the crystalline-growth rate (G) was calculated from the radial crystal growth and expressed in µm/min.

#### 2.3.5. Dynamic Mechanical Analysis (DMA)

The DMA experiments were carried out with a Discovery DHR-1 oscillatory rheometer (TA Instruments, New Castle, DE, USA) equipped with a clamp system for solid samples in torsion mode. Film samples of a 12 mm × 40 mm size were subjected to a heating program from −20 °C to 185 °C (175 °C for PHBH), with a heating rate of 2 °C/min at a constant frequency of 1 Hz. The maximum deformation (*γ*) was set to 0.1%.

#### 2.3.6. Thermogravimetric Analysis (TGA)

The TGA measurements of the neat PHAs and their blends were carried out using the extruded films with a TG-STDA Mettler-Toledo analyzer, specifically the TGA/STDA851e/LF/1600 model (Columbus, OH, USA). The samples with an initial mass of, typically, about 15–17 mg were heated from 25 °C to 900 °C at a heating rate of 10 °C/min under 50 mL/min air flow. The thermal stability of samples was evaluated and the residue at 600 °C was determined. The onset decomposition temperature (T_5%_, temperature at 5% weight loss) and the maximum decomposition rate temperature (T_max.d_) were determined from the weight loss curve and the maximum value of weight loss derivative, respectively.

#### 2.3.7. Mechanical Properties and Tear Resistance

The specimens for mechanical properties were cut in the machine direction (MD) and tested at 0 days and 15 days. Tensile tests were carried out in accordance with the ASTM D638 standard in a universal testing machine, equipped with a 500-N load cell (Shimadzu AGS-X, Kyoto, Japan) at room temperature with a cross-head speed of 10 mm/min. Samples were previously die-cut into type IV specimens (width 6 mm, length 55 mm) after extrusion. Tear-resistance tests were carried out in accordance with the ISO 6383-1 standard into trouser-shaped specimens (width 50 mm, length 100 mm) in the same universal testing machine, at a speed of 200 mm/min. To prevent ageing in samples before testing, half of the specimens for tensile and tear tests were stored at −40 °C before their characterization. These were labeled as *0 days*. In contrast, the rest were stored in a desiccator at room temperature for *15 days*, to allow physical ageing [43,44], and were subsequently used for testing. A minimum of six specimens were measured for each test, time, and sample, and the average results with standard deviation were assessed.

#### 2.3.8. Thermoforming

A vacuum-assisted thermoforming study was carried out in an SB 53c pilot plant (Illig, Helmut Roegele, Germany), equipped with an infrared-emitter heating device. The mold used was a male cylinder tray with a diameter and height of 55 mm and 15 mm, respectively. Sheets of 15–17 cm long and 400 µm thick were cut and preserved until testing as previously mentioned specimens (see Section 2.3.7).

The sheets were stamped with a square grid pattern (2 mm × 2 mm) in order to track deformation during their mold conformation. For all of the experiments, the infrared heater was set to 600 °C, while the heating time was changed in order to indirectly control the temperature of the PHA sheet. The thermoformability was evaluated in accordance with the methodology proposed in a previous work of the group [19].

**Figure 2:**
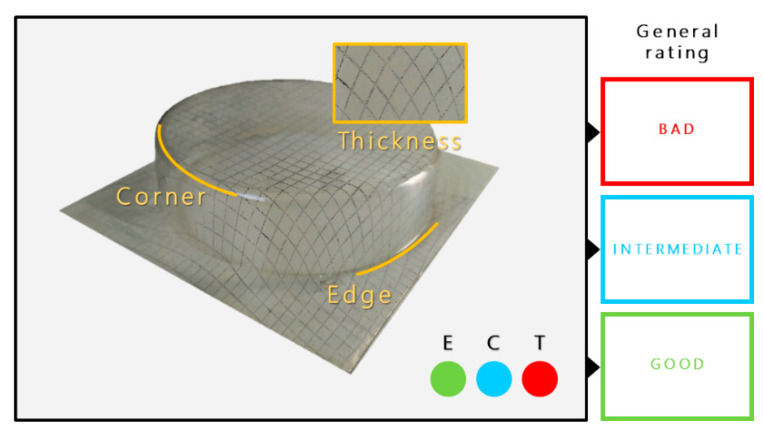
Criteria for the evaluation of thermoformed trays using a positive mold: Linearity of junctions (E), curvature of rounded corner (C), and widening of stamped square net (T). The three parameters lead to a general evaluation for each time shown with the color of the frame.

Briefly, this methodology (see Figure 2) allows the quality of the thermoforming to be classified in accordance with three parts of the thermoformed trays: the *edge*, *corner*, and *thickness distribution*. Each one was classified as *bad* (red color), *intermediate* (blue color), or *good* (green color). This classification was used to establish the thermoforming time windows for each composition.

#### 2.3.9. Melt-Flow Index (MFI)

The MFI values were measured in a melt-flow indexer, model BMF-001.02 (Zwick Roell, Ulm, Germany), according to ISO 1133 standard. The tests were carried out at 180 °C and 2.16 kg load.

#### 2.3.10. Marine Biodegradation

Prior to starting the biodegradation tests, the PHA pellets were powdered by cryogenic milling under liquid nitrogen and subsequent sieving through a 250 µm mesh. Marine biodegradation was determined following the method described in the ASTM D6691 standard, for the first 60 days of testing. Airtight topaz 125-mL bottles were filled with 75 mL of autoclaved natural seawater from Calpe (Alicante, Spain), enriched with 0.5 g/L of NH_4_Cl and 0.1 g/L of KH_2_PO_4_. Then, the solutions were mixed with 20 mg of powdered sample and inoculated with 250 µL of a concentrated marine-bacteria inoculum. All of the samples studied, cellulose (reference) and a blank were prepared in triplicate. Bioreactors were kept at 26 °C and a rotation of 50 rpm for 2 months in an orbital shaking incubator model 2102 (Comecta, Barcelona, Spain). Aerobic conditions were guaranteed throughout the entire experiment. Carbon dioxide evolved by microorganisms was registered by a G110 IR analyser (Fonotest, Madrid, Spain). The percentage of aerobic biodegradation (*B*%) was calculated by comparing the accumulated CO_2_ at a specific time with the total theoretical carbon dioxide using Equation (2):

(2) $$B\;\left(\%\right)=\left(\frac{CO_{2}\left(t\right)-CO_{2}\left(b\right)}{ThCO_{2}}\right)\cdot 100$$

where *CO*_2_ (*t*) is the accumulated carbon dioxide of the sample at a specific time, *CO*_2_ (*b*) is the average accumulated carbon dioxide of the blank at the same time, and *ThCO_2_* represents the total theoretical carbon dioxide calculated from the total organic carbon and the mass of each sample.

## 3. Results

### 3.1. Morphology

The SEM analysis was carried out on the cross-section of films in order to analyze the morphology and phase distribution of the PHA blends. Micrographs were taken in both MD and TD to assess the film orientation during cast extrusion. SEM micrographs showed a smooth uniform surface at any magnification (from 500x to 5000x), displaying the presence of nucleating agents from the commercial PHBV and PHBH samples. Regarding the blending compositions, no remarkable differences were observed with the addition of PHBH to the PHBV matrix.

**Figure 3:**
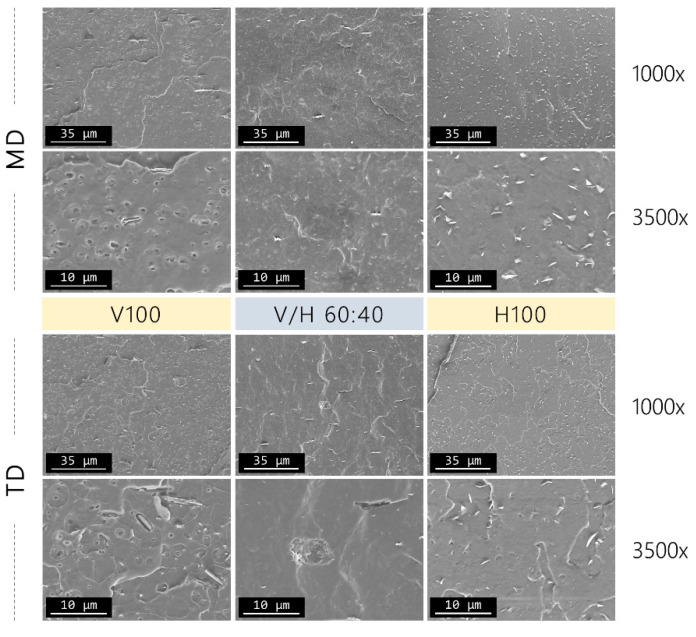
Scanning electron microscopy (SEM) micrographs of neat poly(3-hydroxybutyrate-*co*-3-valerate) (PHBV) and poly(3-hydroxybutyrate-*co*-3-hydroxyhexanoate) (PHBH) and their blend V/H 60:40 at two different magnifications per extrusion direction. Machine direction (MD), up. Transversal direction (TD), down.

As an example, representative SEM images of the V/H 60:40 blend are shown in Figure 3. Even this blend is based on the highest content of PHBH, as it exhibited a monophasic structure regardless the extrusion direction. This suggests good miscibility of the biopolyesters throughout all composition ratios studied.

### 3.2. Crystallization Studies

#### 3.2.1. Crystallization Kinetics

A polarized optical microscope equipped with a hot-melt stage was used to observe the effect of the presence of the low-crystalline PHBH on the spherulite morphology and the crystallization rates of PHBV.

**Figure 4:**
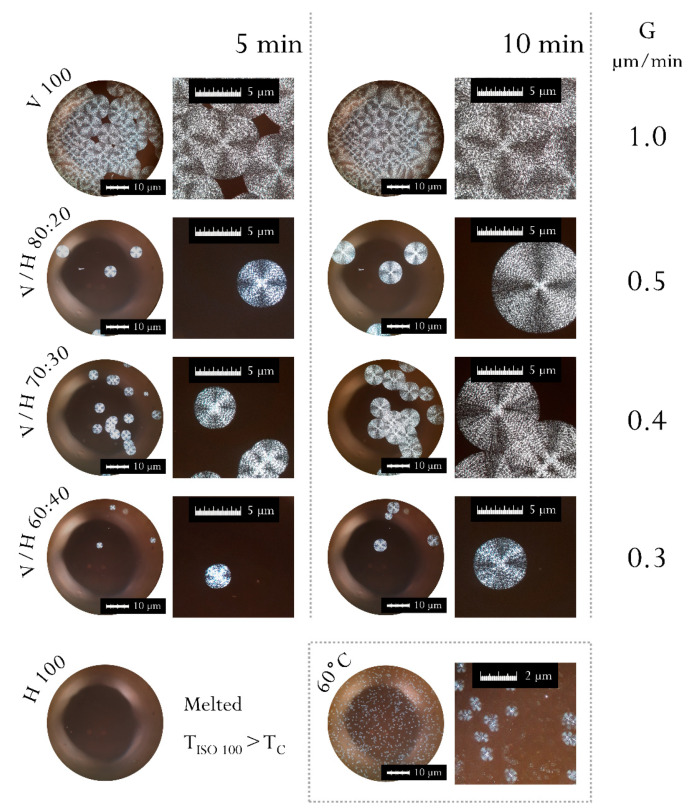
Spherulitic morphology and crystal size viewed through a polarized filter of poly(3-hydroxybutyrate-*co*-3-valerate) (PHBV), poly(3-hydroxybutyrate-*co*-3-hydroxyhexanoate) (PHBH), and their blends. Crystals isothermally formed at 100 °C at two different times. Circular images at 10x, squared images at 10x plus 4x digital magnifications.

Crystallization kinetics in copolymers are complex and involve a great variety of phenomena, such as the production of primary nuclei, the interdiffusion of crystallizable and non-crystallizable chains, or the presence of nucleating agents [45]. In order to remove any possible impurity and the nucleating agents, samples were filtered before casting upon the microscope coverslip. An initial heating step at 190 °C for 2 min was applied to all of the cast samples to ensure the full melting of the materials. Laycock et al. [46] determined this time and temperature as the minimum necessary to avoid the presence of unmelted microcrystals of PHA, while limiting thermal degradation by the melt time. Molten samples were quenched to 100 °C to promote the crystallization of the biopolyesters. After 15 min of isothermal crystallization at 100 °C, the crystalline growth rate, G, was calculated and its values are given in Figure 4.

A decrease in the size of the spherulites for equivalent crystallization times was observed for the blends as the PHBH content increased. As a consequence, the growth rate decayed from 1 µm/min for the neat PHBV to 0.5 µm/min, 0.4 µm/min, and 0.3 µm/min for the samples with 20% wt., 30% wt., and 40% wt. of PHBH, respectively. This led to a drop of 50% in the crystallization speed, with the addition of 20% wt. of PHBH to the blend. Nonetheless, the subsequent additions of 10% wt. of PHBH until a 60:40 ratio (PHBV/PHBH) only supposed a reduction of 0.1 µm/min each. As other authors have mentioned, the non-crystallizing or slow-crystallizing component may be excluded from the growing spherulites, thus being found in excess at the spherulitic boundaries [46]. As a result of the exclusion of voluminous 3HH units from the crystalline phase, the diffusion of 3HB and 3HV units to crystals is hindered by the progressive addition of bulky, non-crystallizable PHBH molecules to the blends. These results are consistent with the results obtained by Chang et al. [47] in binary blends of PHBV (semi-crystalline) and PVAc (amorphous).

Regarding the morphology, the spherulites showed the typical ring-banded patterns and the Maltese-cross of random copolymers (Figure 4). The Maltese-cross pattern, presented in all of the samples studied, originates from a parallel or perpendicular crystal axis with respect to the polarization direction [48]. The banded structures have been associated to the presence of periodically twisted lamellar crystals coming from the release of chain-conformation stresses [49].

In this regard, Hsieh et al. [47,50] found that, in PHBV/PVAc blends, the regularity of PHBV rings becomes better and the band spacing decreases significantly with the increasing content of the amorphous polymer. The authors proposed that the additional excess surface stresses, induced by the interaction between the amorphous phase and the lamellar crystals, increased the twisting of lamellae. Contrary to what was postulated, and with the exception of unbanded neat PHBV, all PHBV/PHBH blends exhibited ring-banded spherulites, which slightly but clearly increased band spacing when higher PHBH (mainly amorphous) contents were used.

#### 3.2.2. Thermal and Crystallization Behavior

DSC experiments were carried out on the neat PHAs and their blends in order to study the melting and crystallization process. The values of ∆H_m_, ∆H_c_, T_m_, T_c_, and ∆H_m.N_ are summarized in Table 2. The melting points of V100 and H100 were consistent with the values reported in the literature: 170–175 °C for PHBV [51] and 114–120 °C for PHBH [52]. PHBV and PHBH exhibited considerable differences in terms of their characteristic melting and crystallization temperatures, that is, 54 °C and 38 °C for T_m_ and T_c_, respectively, with both being higher for PHBV. In a general manner, PHBV/PHBH blends exhibited an intermediate thermal behavior to both neat components but closer to that of PHBV.

**Figure 5:**
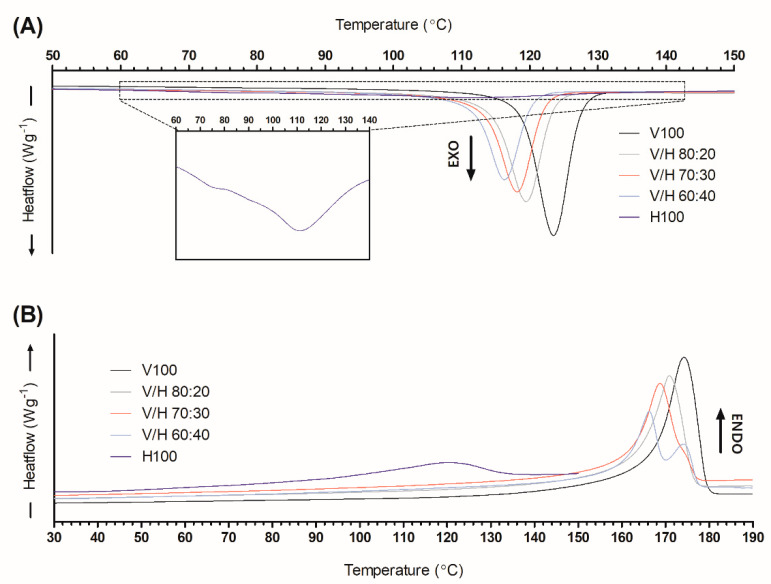
Cooling (**A**) and second-heating (**B**) curves of poly(3-hydroxybutyrate-*co*-3-valerate) (PHBV), poly(3-hydroxybutyrate-*co*-3-hydroxyhexanoate) (PHBH), and their blends with PHBH crystallization curve detailed.

Figure 5 plots the representative thermograms of the cooling and second heating steps for all of the materials studied. Regarding crystallization behavior, as observed from the cooling curve (Figure 5A), PHBV and its blends showed a single peak of crystallization, while PHBH presented a wider and less intense peak with a little shoulder at lower temperatures. This behavior indicates that two populations of crystals were generated during cooling or could result from a melt–recrystallization phenomenon [53,54]. However, this last phenomenon is more representative of MCL-PHAs with a side-chain length that is greater than C4 [55]. Additionally, the T_c_ values of blends were shifted towards lower temperatures as the PHBH content increased. This may indicate that the presence of PHBH hinders the crystallization process of PHBV crystals, which is in accordance with POM observations.

As pointed out by Noda et al. [56], the incorporation of different MCL-PHAs into PHB decreased its melting point due to the reduction in packing efficiency in the crystals. Regarding the melting behavior, as evaluated from the second-heating curve (Figure 5B), the PHBV and low-PHBH-content blends presented a single melting peak. Similar to the crystallization curves, the melting peak temperature decreased with the PHBH addition. For the 40% wt. PHBH blend, the melting event occurred in two melting peaks. Multiple melting peaks are generally ascribed to melting, recrystallization, and remelting phenomena due to the annealing undergone by the material during heating [57]. In this case, the first peak, seen at 166 °C, would be the melting temperature corresponding to the crystals generated during cooling, which agrees with the trend observed in the other blends [53]. The progressive decrease in the melting temperature of PHBV with the addition of PHBH could be explained by a reduction in the size of the crystalline domains, which was potentially induced by the steric hindrance imposed by PHBH onto the crystallization of the PHBV chains [52].

**Figure 6:**
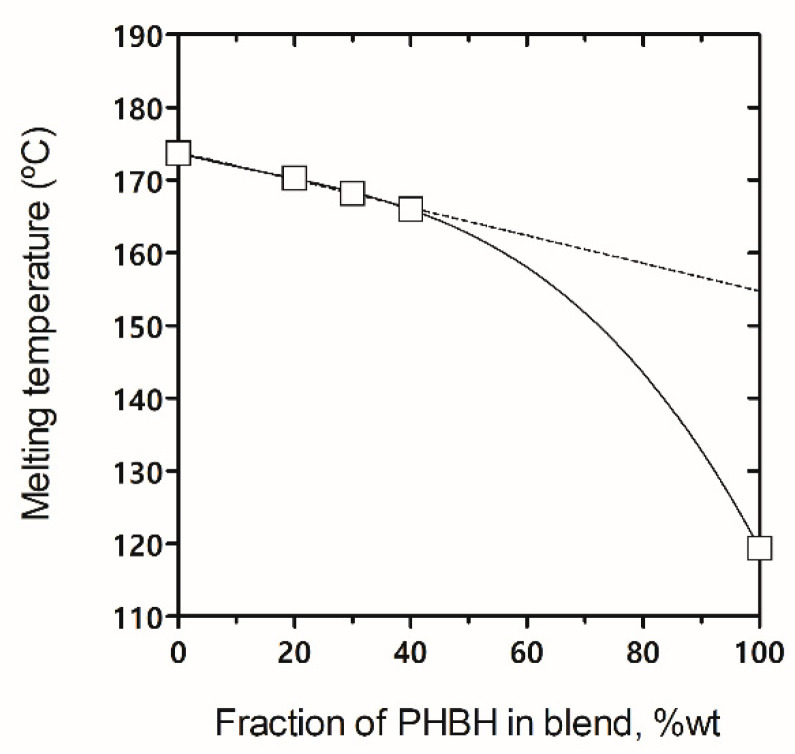
Melting temperature values of the poly(3-hydroxybutyrate-*co*-3-valerate) (PHBV)/poly(3-hydroxybutyrate-*co*-3-hydroxyhexanoate) (PHBH) blends depending on the PHBH fraction.

As can be observed in the normalized enthalpies, also shown in Table 2, no significant differences were seen with respect to the neat PHBV melting enthalpy or between the blends. This may indicate that only PHBV crystallized, while PHBH remained in the amorphous fraction. Therefore, although the crystalline PHBV domains were smaller in the blends than in the neat material (as observed by both DSC and POM), it is confirmed that the degree of crystallization of PHBV in the blends remained nearly the same as in the pristine PHBV. Similarly, Yoshie et al. [58] found that PHBP chains could not participate in the PHB-type lattice, so only the homogeneous sequences of 3HB units longer than the lamellas could participate in the lamellas induced by PHBV. Thus, PHBP hardly co-crystallized with PHBV in the PHBV/PHBP blends. Moreover, according to the T_m_ patterns described by Yoshi et al. [59], melting points as a function of the PHBH fraction in the blend were plotted in Figure 6 to discern phase structures in the blends.

The melting point of neat PHBH did not follow a linear correlation with the T_m_ of PHBV and their blends. Although it would be convenient to complete the curve with PHBH fractions > 50% wt., it is presumable that a straight line would deviate into a convex curve. These data suggest the miscibility of the amorphous phase and partial segregation of the crystalline phase, during melting with PHBV-rich crystals [60,61].

#### 3.2.3. Crystal Structure

**Figure 7:**
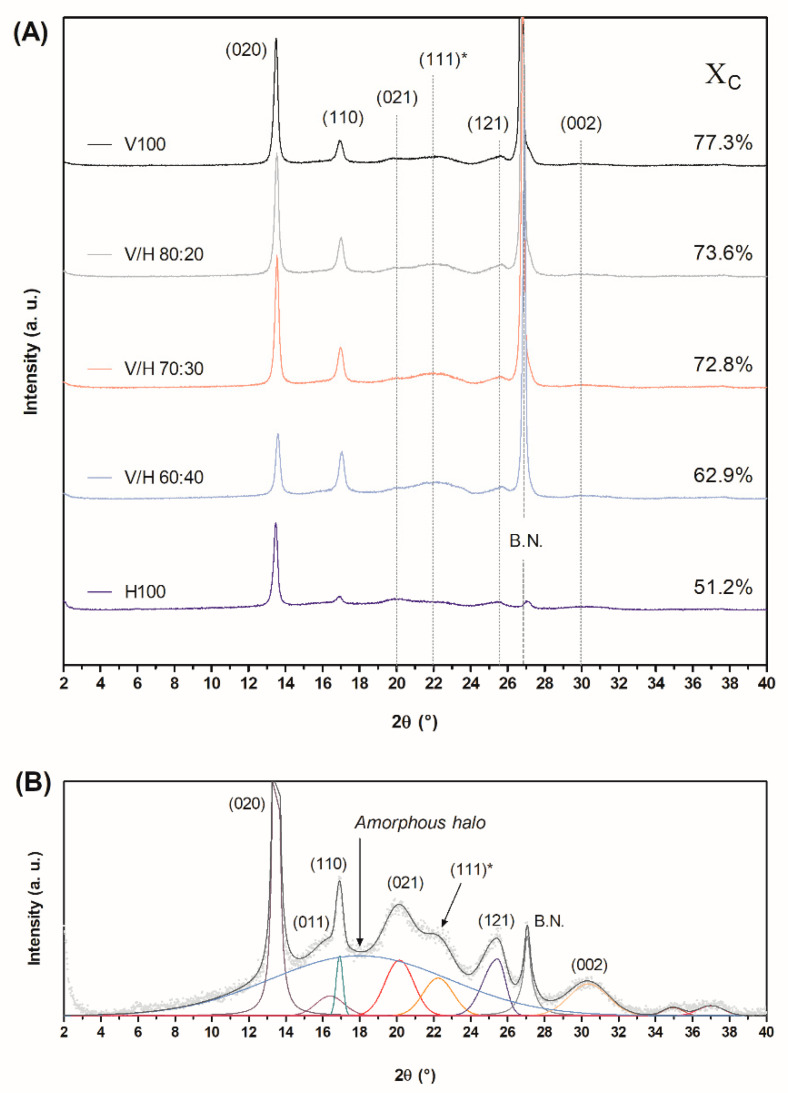
(**A**) X-ray-diffraction patterns of the poly(3-hydroxybutyrate-*co*-3-valerate) (PHBV), poly(3-hydroxybutyrate-*co*-3-hydroxyhexanoate) (PHBH), and their blends including the degree of crystallinity (X_C_) on the right; (**B**) Peak deconvolution of neat PHBH diffractogram with a detailed amorphous halo.

Wide-angle X-ray scattering was carried out in order to study the crystalline-structure changes with the addition of PHBH to the PHBV matrix. The diffractograms and crystalline degree of the biopolymers and their blends are shown in Figure 7.

Except for PHBH, the most intense peak at 27° (2*θ*) belongs to boron nitride, incorporated as a nucleating agent in these commercial polymers. PHBV yielded two main peaks at 13.5 and 17° (2*θ*), which are associated with the (020) and (110) reflections of the orthorhombic lattice of PHBV, respectively [62]. PHBH crystallized in a PHB orthorhombic lattice [63] and showed the same two peaks associated with the same planes, but the second one was much less intense. In the case of PHBH, the rest of its characteristic peaks were also almost hidden by the amorphous halo. Thus, for all the blend compositions, this halo centered at 18° (2*θ*) and associated with the amorphous fraction of the material, which increased as the PHBH fraction in the blend increased.

No noticeable differences in the peak positions were detected for any blend. It suggests that the basic crystal structure of PHBV (the crystal lattice) remained unaltered. However, there were some relative intensity changes when PHBH was incorporated into PHBV. Hence, as the PHBH content increased, the intensity of the (020) peaks decreased while the (110) and (111)* peaks increased. In pristine PHBH, these last two peaks are difficult to appreciate. Moreover, plane (021) was more intense than (111)* in H100. In particular, (111)* represents the fusion of planes (111) and (101) in just one wider peak, due to the processing temperature that changes the crystalline domains of 3HB and 3HV, as reported by Bossu et al. [64] for PHBV with 3 % mol. 3HV and 18 % mol. 3HV. Intensity-ratio variation of main peaks (020) and (110) can be attributed to the different size or the different reorganization rates of the planes during the lamellar-thickening process through the interlamellar slip [65].

According to the literature, PHBV and PHBH both crystallized in the PHB lattice [63,66], but in different manners. PHBV is well-known to have an isomorphous co-crystallization behavior, depending on the major component of the copolymer. Hence, for PHBV with 3HV contents below 30 % mol., 3HV units are included in the PHB lattice. On the other hand, other PHB copolymers with a higher number of carbons in the monomeric unit do not show isomorphous behavior [58]. This is the case of PHBH, where bulky 3HH units are excluded from the crystal lattice, while the 3HV monomer is incorporated into the PHB crystal in looser structures [67], thus rendering a lower degree of crystallization, 51.2%.

Compared to PHBV, a difference of approximately 26% in the degree of crystallinity was observed between the neat biopolymers. The theoretical degree of the crystallinity of blends based on X_C_ and the weight fraction of each PHA only differed by 1.5%, 3%, and 4% (V/H 80:20, 70:30, and 60:40, respectively) with their experimental X_C_. Thus, in accordance with the DSC results, PHBH hinders crystal formation and crystalline domains as well as reduces the kinetics of PHBV, but it does not avoid the final degree of crystallinity in the blends.

### 3.3. Dynamic Mechanical Analysis

**Figure 8:**
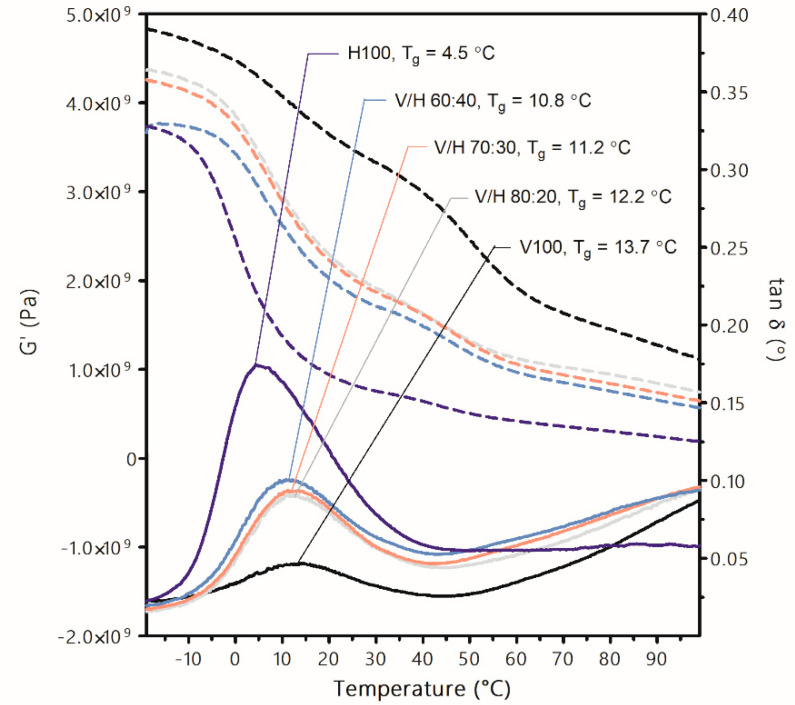
Storage modulus (G’, left axis, discontinuous lines) and tan δ values (right axis, continuous lines) of poly(3-hydroxybutyrate-*co*-3-valerate) (PHBV), poly(3-hydroxybutyrate-*co*-3-hydroxyhexanoate) (PHBH), and their blends. Glass transition temperatures (T_g_) are also indicated.

The viscoelastic behavior of the PHA blends was studied by means of DMA. Figure 8 displays the storage modulus and tan *δ* curves from −20 °C to 100 °C. All blends exhibited a single peak in the tan *δ* curve, this being a relaxation attributed to the glass transition of the material [68]. The presence of a single peak in the blends confirms the full miscibility of the PHAs in the amorphous fraction, in agreement with previous observations. In addition, the T_g_ values shifted to lower temperatures as the PHBH content increased [23].

PHBH showed a more intense relaxation at 4.5 °C than neat PHBV with a T_g_ at 13.7 °C, this being the result of a major presence of the amorphous fraction in PHBH when compared with the highly crystalline PHBV [68]. The G’ and tan *δ* curves of the blends showed an intermediate behavior between the neat PHAs, both in terms of the drop in the storage modulus and intensities of the relaxation, respectively, as the PHBH content increased. Despite the variation in the viscoelastic behavior of PHBV when PHBH is incorporated, the changes were not proportional to the blend composition. For instance, the addition of 40% wt. of PHBH only generated a reduction of 3 °C in the tan *δ* peak.

The G’ modulus, which is a measure of elastic behavior, is associated with stiffness and is conceptually related to Young’s modulus [69]. In this regard, mechanical performance in terms of stiffness dependence with temperature can be inferred. The value of G’ was mostly affected by the presence of PHBH, but not directly proportional to the MCL-PHA present in the blend.

### 3.4. Thermal Stability

**Figure 9:**
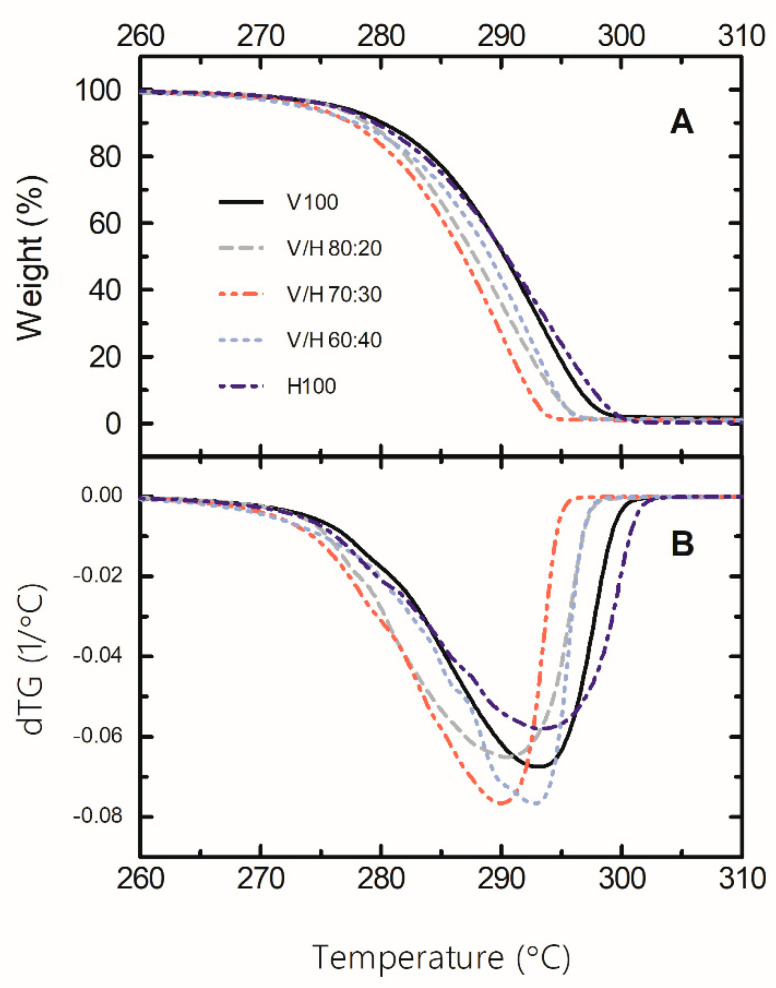
Thermogravimetrical analysis (TGA) (**A**) and derivative thermogravimetric (DTG) (**B**) curves of poly(3-hydroxybutyrate-*co*-3-valerate) (PHBV), poly(3-hydroxybutyrate-*co*-3-hydroxyhexanoate) (PHBH), and their blends.

The thermal stability of PHBV, PHBH, and their blends was assessed by TGA. The corresponding TGA and derivative thermogravimetric (DTG) curves are shown in Figure 9. The T_5%_ and T_max.d_ values are displayed in Table 2. The TGA curves showed that the thermal degradation of PHBV and PHBH occurs in a single step process. According to the literature [70], a random chain scission by means of a cis-elimination mechanism, also called the McLafferty rearrangement, is considered as the general pathway of thermal degradation for PHB and PHBV.

PHBH, which differs from PHBV in the length of the side chains, followed the same one-step process with almost the equivalent maximum decomposition temperature, at around 293 °C. This agrees with Lee et al. [71] in their report on the thermal-degradation mechanism for PHO, another MCL-PHA similar to PHBH. The blends exhibited a very slight decrease in T_5%_ and T_max.d_, which may suggest a certain decrease in molecular weight during processing. In this regard, it is worth mentioning that the blends were subjected to an additional thermal treatment (melt blending) compared to the neat PHAs that were used as received for cast extrusion. Particularly, as the PHBH content increased, the thermal stability of the blend system increased. One can consider that the side chains of MCL-PHAs sterically hindered the formation of the six-membered transition structure, resulting in a higher thermal degradation temperature [51]. However, in general, the TGA parameters were similar to those of the neat copolymers, 3 °C being the maximum difference between them. Therefore, it can be concluded that the blends are thermally as stable as the neat components.

### 3.5. Mechanical Behaviour

**Figure 10:**
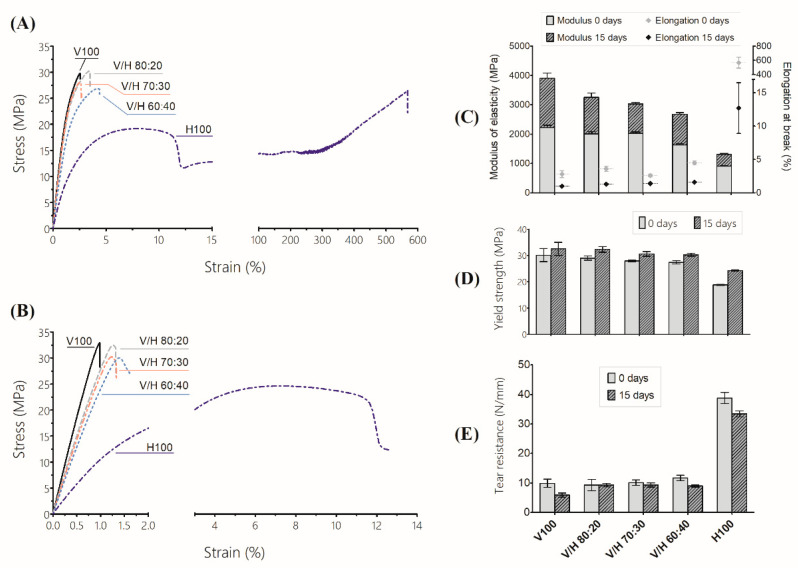
Mechanical properties of poly(3-hydroxybutyrate-*co*-3-valerate) (PHBV), poly(3-hydroxybutyrate-*co*-3-hydroxyhexanoate) (PHBH), and their blends: (**A**) Representative stress–strain curves at 0 days; (**B**) Representative stress–strain curves at 15 days; (**C**) Modulus of elasticity (bar, left axis) and elongation at break (dot, right axis); (**D**) Yield strength; (**E**) Tear resistance.

The mechanical behavior of PHBV, PHBH, and their blends was studied by tensile test, up to failure and tear resistance. The tensile modulus of the elasticity €, yield strength (*σ*_y_), and percent of elongation at break (*ε*_b_) of the different compositions and times (0 days and 15 days) in MD are shown in Figure 10. Representative strain–stress curves of neat polymers and blends at 0 days and 15 days are also displayed.

Crystallization behavior plays a major role in the mechanical properties of PHAs. Crystallinity has a direct effect on tensile modulus and tensile strength [72]. Thus, highly crystalline PHAs, such as PHBV with low 3HV content, exhibit an intrinsic fragile character, this brittleness being dependent on the developed crystallinity [18,72]. Furthermore, PHAs suffer a further progressive embrittlement over time, which is attributed to a combination of two phenomena: a secondary crystallization process and physical ageing of the amorphous phase when T > T_g_ [16].

On the other hand, PHBH presented a typical strain–stress curve of a ductile polymer, as derived from its low crystallinity. Nevertheless, the change undergone by the tensile properties over time follows a similar trend to that of PHBV, showing an increase in the modulus of elasticity and yield strength, with a great reduction in elongation at break. The addition of PHBH to PHBV in the blends resulted in an intermediate behavior in terms of E and *σ*_y_. However, *ε*_b_ was barely affected by the addition of PHBH. These trends can be explained by assuming that the crystalline fraction of the SCL-PHA is ruling the mechanical properties of the blends (similar to neat PHBV).

Hence, the modulus of elasticity decreased when increasing the PHBH content in the blends. This trend was clearer after 15 days, when the crystallinity was almost fully developed. As mentioned in Section 3.2, although a small addition of PHBH greatly changed the crystallization rate, the final degree of crystallinity of PHBV did not change significantly. Yield strength in PHBV and its blends were not affected as much as it was with neat PHBH. As all of these samples showed brittle behavior, so this stress may be related to a toughness value rather than with an elastic-to-plastic transition.

Ductility seemed to be the parameter most sensitive to ageing. For all the blends studied, the intrinsic brittleness of the crystalline fraction of PHBV clearly dominated the behavior of any composition. Even in the blend containing 40% wt. of PHBH, the contribution of PHBH slightly increased the *ε*_b_ at 0 days, from 2.8% for the neat PHBV to 4.5%. The decrease in *ε*_b_ after 15 days was remarkable for all the materials studied, dropping to 1%–1.5% for PHBV and the blends, and to 12.7% for neat PHBH. Secondary crystallization and the physical ageing of the amorphous fraction trapped between lamellae are considered to constrict the structure of and around the spherulites [16]. The boundaries and internal fissures of spherulites could act as stress concentrators that promote fracture. Thus, the slight effect of the ductile biopolymer in terms of the toughening capacity, despite the increased mobility of the amorphous fraction, can be attributed to the fact that the fracture of PHBV is dominated by the crystal fraction, as the initial steps of the fracture take place in the internal flaws between the lamellae that easily become critical within the same spherulite [73].

Figure 10E summarizes the results of the average tear resistance, obtained by means of the tearing test of the trouser method. In agreement with the tensile-test results, the tear resistance follows the same tendency as ductility. PHBV presented a low tear resistance, with values of less than 10 N/mm for 0 days, decreasing to 6 N/mm after 15 days. On the other hand, PHBH showed a high tear-resistance value, around 39 N/mm, which underwent a slight decrease (below 15%) with the ageing time. With respect to the blends, all of the compositions studied presented values from 10 N/mm to 12 N/mm, regardless of the ageing time. These results are in the range of the neat PHBV, thus confirming the low toughness of the blends. However, the incorporation of PHBH seems to avoid the effect of ageing. Moreover, for each studied formulation and from a practical point of view, it can be considered that ageing barely impacts tear resistance.

### 3.6. Thermoforming Studies

**Figure 11:**
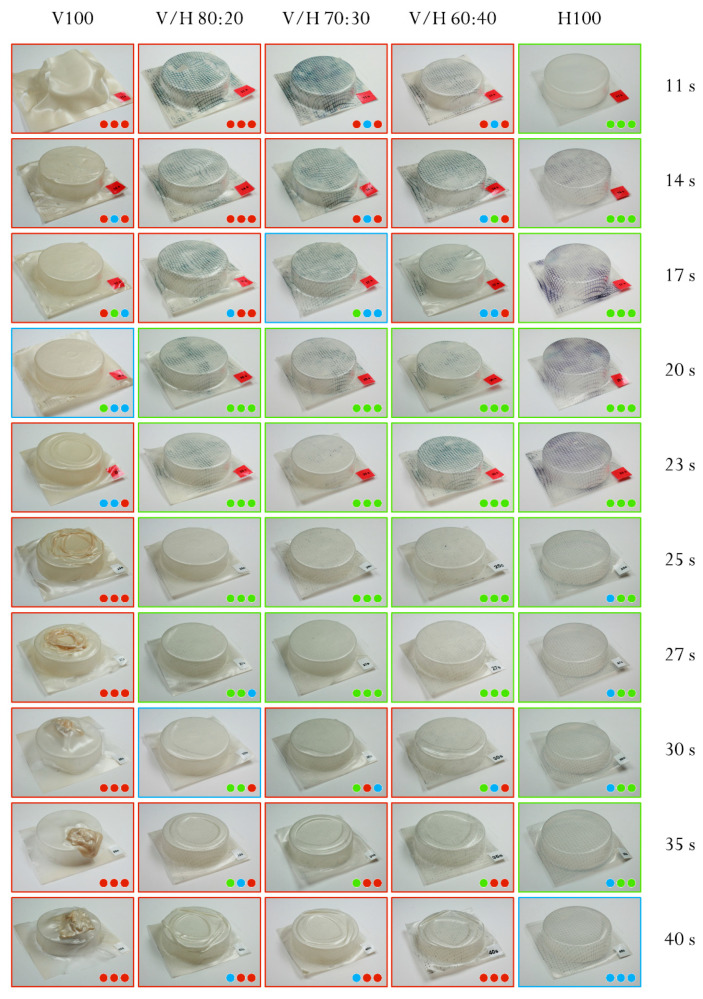
Photographs and evaluation of the thermoformed structures of poly(3-hydroxybutyrate-*co*-3-valerate) (PHBV), poly(3-hydroxybutyrate-*co*-3-hydroxyhexanoate) (PHBH), and their blends depending on the processing time. Each image is accompanied by three assessing symbols in order of edge–corner thickness from left to right.

Thermoforming is a low-cost processing technology widely used in the plastics industry to transform polymer sheets into a great variety of shapes, mainly trays and blisters. PHBV is known for having a poor thermoformability due to a high crystallinity and low melt strength, hence, resulting in a very narrow processing window. Their adequate softening temperature is close to the melting point of the polymers, thus reducing the operating temperature range [19]. In fact, for the neat PHBV, none of the studied operational conditions (from 11 s to 40 s of heating time) led to a good thermoformed tray, as shown in Figure 11. On the one hand, at a low heating time (i.e., lower temperatures) the sheet was not fully plasticized. Therefore, the stiffness of the polymer did not allow the sheet to yield in order to replicate the shape of the mold accurately. On the other hand, at a high heating time (i.e., high temperatures), the sheet underwent a sagging effect [74] (related to a sudden loss in the mechanical stability of polymer) leading to an uneven distribution of thicknesses that was seen at heating times above 23 s.

Conversely, neat PHBH presented a good thermoformability throughout the heating range studied. This can be ascribed to a higher melt elasticity [75], associated with a lower crystallinity and a mechanical behavior governed by the predominant amorphous fraction. Regarding the blends, for all the compositions studied, a good thermoformability with an extended processing window was detected, as can be observed in the images. Furthermore, the amount of PHBH in the blend composition did not appear to have a relevant effect on thermoformability, the results being similar for all of the blends studied: the processing window has been widened from 20 s to 27 s (*green* color frame). The addition of PHBH resulted in all cases in an increase in the melt elasticity, thus rendering an improved mold reproduction together with a decrease in the sagging effect.

This general improvement and undifferentiated results for all blends agree with the MFI values obtained (see Table 3). The MFI values dropped drastically with the addition of any percentage of PHBH and were constant for all of the blends.

### 3.7. Marine Biodegradability

**Figure 12:**
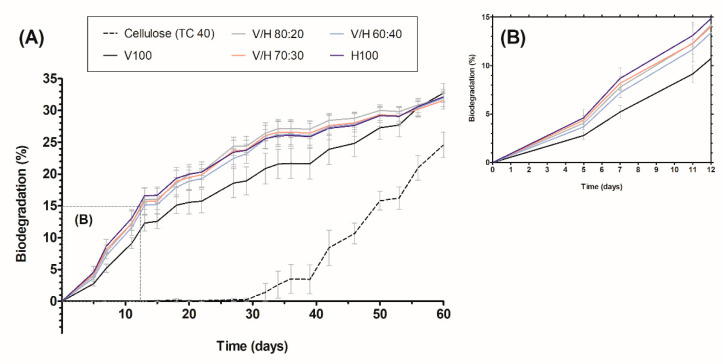
(**A**) Percentage of biodegradation over time of poly(3-hydroxybutyrate-*co*-3-valerate) (PHBV), poly(3-hydroxybutyrate-*co*-3-hydroxyhexanoate) (PHBH), their blends, and the reference (cellulose); (**B**) Detail of first 12 days of marine biodegradation.

Biodegradation is a complex process that depends on many different factors, such as the chemical composition, structure, or crystallinity of the materials as well as the temperature and microbiome (fungi, yeast, bacteria) of the tested environment [76]. Following ASTM D 6691, the natural seawater-inoculum option, biodegradation of PHBV, PHBH, and their blends were assessed in a simulated marine environment during the first two months of testing. The evolution of biodegradation (B%) over time is shown in Figure 12.

All compositions clearly exceed the cellulose biodegradation during all the tests. This can be attributed to the low cellulolytic capability of the inoculum, which is also responsible for the initial lag of almost 30 days for the onset of cellulose biodegradation.

In this context, one should take into account that PHAs are known to be fully biodegradable in all kinds of environments, including marine [77]. However, the higher amorphous fraction of PHBH resulted in a higher biodegradation rate compared to the more crystalline PHBV, with a curve that was kept approximately 3% lower until day 50. The amorphous fraction is preferred by microorganisms to be degraded first in the initial stages because the mobility of the chains facilitates the accessibility of their enzymes [78]. As can be seen in Figure 12B, the biodegradation rate of the blends was slightly different, but all curves were located between the neat biopolymers according to the variation of crystallinity. Despite the different rate of biodegradation, the final degree of biodegradation reached by all of the tested samples stabilized around 32% after 60 days, as can be seen in Figure 12A. These results agree with the 30% of biodegradation reported by Meereboer et al. [79] for PHBV by day 60, following the standard ASTM D 7991.

## 4. Conclusions

Blends of PHBV with a content of increasing PHBH, of up to 40% wt., have been obtained by melt-mixing and subsequent cast extrusion. The influence of the addition of the MCL-PHA to PHBV on the morphology, crystallinity, thermal and mechanical properties, thermoformability, and biodegradability has been studied. In spite of all compositions exhibiting full miscibility (single T_g_ peak), the crystallinity of the resulting blends was limited to the PHBV fraction. Thus, although bulky 3HH units hindered the spherulite growth rate of PHBV from 1.0 µm/min to 0.3 µm/min, the eventual degree of crystallinity achieved by PHBV remained unaltered. The experimental degree of crystallization of blends obtained by WAXS (74%, 73%, and 63% for 20% wt., 30% wt., and 40% wt. of PHBH) only differed by 1.5%–4.0% compared to the theoretical degree of crystallization calculated, according to the weight fraction of the blend components. The crystalline phase still governed the mechanical performance of the blends, thus revealing a poor toughening effect of PHBH for all of the studied range. Hence, the mechanical parameters of PHBV were slightly affected in the blends. However, the incorporation of PHBH successfully resulted in an increase in the melt elasticity, regardless of the amount added, thus leading to an improved thermoformability, which considerably broadened the processing window by 7 s from the non-optimal processing window of PHBV. Biodegradation studies in simulated marine environment confirmed that, after two months, all of the studied compositions reached the same degree of biodegradation (around 32%). Therefore, this study has proven that the direct blending of miscible MCL-PHA nearly improves the mechanical performance of low-3HV content PHBV since it is still governed by the intrinsically brittle crystalline nature of the SCL-PHA. However, it does represent an effective approach to enhancing its processability by means of thermoforming.

## Figures and Tables

**Table 1:** Composition and nomenclature of the compositions according to the weight content (% wt.) of poly(3-hydroxybutyrate-*co*-3-valerate) (PHBV) and poly(3-hydroxybutyrate-*co*-3-hydroxyhexanoate) (PHBH).

Sample Code	PHBV (% wt.)	PHBH (% wt.)
V100	100	0
V/H 80:20	80	20
V/H 70:30	70	30
V/H 60:40	60	40
H100	0	100

**Table 2:** Thermogravimetrical analysis (TGA) and differential scanning calorimetry (DSC) data for poly(3-hydroxybutyrate-*co*-3-valerate) (PHBV), poly(3-hydroxybutyrate-*co*-3-hydroxyhexanoate) (PHBH), and their blends.

	TGA Parameters			DSC Parameters				
	T_5%_ (°C)	T_max.d_ (°C)	R_600_ (%)	T_m_ (°C)	∆H_m_ (J/g)	∆H_m.N_ (J/g)	T_c_ (°C)	∆H_c_ (J/g)
V100	276.3	293.0	0.8	173.7	94.7	94.7	124.2	83.1
V/H 80:20	276.0	290.5	0.7	170.3	76.5	95.6	120.1	66.7
V/H 70:30	274.0	290.0	0.5	168.2	67.7	96.6	118.7	60.8
V/H 60:40	273.3	292.8	0.3	166.0/173.9	56.4	93.9	116.8	52.2
H100	276.0	293.3	0.2	119.4	26.5	-	86.2	29.8

**Table 3:** Melt-flow index (MFI) of poly(3-hydroxybutyrate-*co*-3-valerate) (PHBV), poly(3-hydroxybutyrate-*co*-3-hydroxyhexanoate) (PHBH), and their blends at 180 °C and 2.16 kg of load.

	V100	V/H 80:20	V/H 70:30	V/H 60:40	H100
MFI (g/10 min)	27.4 ± 4.4	7.3 ± 0.3	7.5 ± 0.3	7.3 ± 0.2	6.2 ± 0.5

## Data Availability

Data is contained within the article and also available on request.

## Abbreviations

The following abbreviations are used in this manuscript:

3HB	3-Hydroxybutyrate units
3HH	3-Hydroxyhexanoate units
3HV	3-Hydroxyvalerate units
EVA	Poly(ethylene-*co*-vinyl acetate)
MCL-PHA	Medium-chain-length polyhydroxyalkanoate
LCL-PHA	Long-chain-length polyhydroxyalkanoate
PBAT	Poly(butylene adipate terephthalate)
PBS	Poly(butylene succinate)
PCL	Poly(3-caprolactone)
PHA	Polyhydroxyalkanoate
PHB	Poly(3-hydroxybutyrate)
P3HB-*co*-4HB	Poly(3-hydroxybutyrate-*co*-4-hydroxybutyrate)
PHBH	Poly(3-hydroxybutyrate-*co*-3-hydroxyhexanoate)
PHBP	Poly(3-hydroxybutyrate-*co*-3-hydroxypropionate)
PHBV	Poly(3-hydroxybutyrate-*co*-3-valerate)
PHO	Poly(3-hydroxyoctanoate)
PMMA	Poly(methyl methacrylate)
PLA	Polylactide
PVA	Poly(vinyl alcohol)
SCL-PHA	Short-chain-length polyhydroxyalkanoate
TGIC	Triglycidyl isocyanurate
TPU	Thermoplastic polyurethane
